# Core Needle Biopsy Targeting the Viable Area of Deep-Sited Dominant Lesion Verified by Color Doppler and/or Contrast-Enhanced Ultrasound Contribute to the Actionable Diagnosis of the Patients Suspicious of Lymphoma

**DOI:** 10.3389/fonc.2020.500153

**Published:** 2020-10-07

**Authors:** Jian Li, Jing Han, Yu Wang, Yunxian Mo, Jibin Li, Jin Xiang, Zhiming Li, Jianhua Zhou, Siyu Wang

**Affiliations:** ^1^Department of Diagnostic & Interventional Ultrasound, Sun Yat-sen University Cancer Center, State Key Laboratory of Oncology in South China, Collaborative Innovation Center for Cancer Medicine, Guangzhou, China; ^2^Department of Medical Oncology, Sun Yat-sen University Cancer Center, State Key Laboratory of Oncology in South China, Collaborative Innovation Center for Cancer Medicine, Guangzhou, China; ^3^Department of Radiology, Sun Yat-sen University Cancer Center, State Key Laboratory of Oncology in South China, Collaborative Innovation Center for Cancer Medicine, Guangzhou, China; ^4^Department of Clinical Research, Sun Yat-sen University Cancer Center, State Key Laboratory of Oncology in South China, Collaborative Innovation Center for Cancer Medicine, Guangzhou, China; ^5^Department of Pathology, Sun Yat-sen University Cancer Center, State Key Laboratory of Oncology in South China, Collaborative Innovation Center for Cancer Medicine, Guangzhou, China; ^6^Department of Thoracic Surgery, Sun Yat-sen University Cancer Center, State Key Laboratory of Oncology in South China, Collaborative Innovation Center for Cancer Medicine, Guangzhou, China

**Keywords:** lymphomas, diagnostic hematology, imaging, color Doppler flow imaging, contrast – enhanced ultrasonography, core needle biopsies

## Abstract

**Background:**

Inadequate accuracy of ultrasound-guided core needle biopsy (US-CNB) urges further improvement for the diagnosis and management of lymphoma to meet with the practitioners’ increased reliance on this mini-invasive approach.

**Methods:**

Data related to US-CNB of the deep-sited dominant lesions suspicious of lymphoma detected by computer tomography or positron-emission tomography/computer tomography for eligibility assessment of three prospective clinical trials were collected in advance. A retrospective analysis of the prospective data collection was performed, in which Viable-targeting US-CNB that Color Doppler flow imaging (CDFI) and/or contrast enhanced ultrasound (CEUS) were employed to select viable area for biopsy target compared with Routine US-CNB that routine procedure of evaluation and guidance using gray-scale ultrasound with CDFI in terms of the yield of clinically actionable diagnosis and safety, and determinants for the successful US-CNB that established an actionable diagnosis were explored. The establishment of final diagnosis was based on surgical pathology or medical response to therapy with follow-up at least 6 months.

**Results:**

A total of 245 patients underwent Routine US-CNB (*N* = 120) or Viable-targeting US-CNB (*N* = 125), of which 91 (91/120, 75.8%) and 112 (112/125, 89.6%) were revealed with actionable diagnoses, respectively (*p* = 0.004, OR 0.846, 95% CI: 0.753–0.952). And 239 patients established final diagnoses. Diagnostic yields of actionable diagnosis according to the final diagnoses were 78.4% (91/116) and 91.1% (112/123) (*p* = 0.006, OR 0.554, 95% CI: 0.333–0.920), 82.6% (90/109) and 92.5% (111/120) for malignancy, 84.0% (84/100) and 91.8% (101/110) for lymphoma, 85.1% (80/94) and 92.3% (96/104) for Non-Hodgkin Lymphoma, 66.7% (4/6) and 83.3% (5/6) for Hodgkin Lymphoma in Routine and Viable-targeting CNB groups, respectively. No major complications were observed. Dominant lesions with actionable diagnosis in US-CNB were with higher FDG-avid Standardized Uptake Value. Binomial logistic regression revealed that actionable diagnosis of US-CNB was correlated with group and ancillary studies.

**Conclusion:**

Viable-Targeting US-CNB was superior to routine US-CNB in term of the yield of actionable diagnosis for deep-sited dominant lesions suspicious of lymphoma, which demonstrated a potential to be the initial approach in this setting.

## Introduction

Lymphoid neoplasms, including Hodgkin lymphoma (HL), mature B cell neoplasms and mature T and NK neoplasms, are malignancies with increasing prevalence involving the young and middle-aged population (1). The estimated new cases and deaths of lymphoma were 88200 and 52100 in China in 2015, and 82310 and 20970 in United States in 2018, respectively (2–4). Its therapeutic management depends strongly on histo-pathologic diagnosis with molecular information as well as the clinical staging (5). To determine the definitive diagnosis of lymphoma, the morphology and immunohistochemistry of resected tumor or biopsy should be reviewed by an experienced pathologist specialized in the diagnosis of lymphoma, and where appropriate, flow cytometry and molecular studies should be necessitated to accurately categorize the lymphoma (1). Surgical incisional or excisional biopsy recommended by National Comprehensive Cancer Network, European Society for Medical Oncology, or World Health Organization, are the preferred methods to obtain adequate tissue for lymphoma diagnosis (1, 5). Core needle biopsy (CNB) is an alternative approach when excisional biopsy is not feasible or to document relapse, and were recommended by Lugano Classification for initial evaluation, staging, and response assessment of Hodgkin and non-Hodgkin lymphoma (5). Fine needle aspiration has been found to be in low accuracy for such diagnosis (6). Concerning biopsy of a lesion suspicious lymphoma located in the deep-sited mediastinum, thoracic, abdominal or pelvic cavity, or retro-peritoneum, CNB is a faster method for obtaining sufficient tumor materials, as compared to surgical procedures, and is more commonly used for lymphoma diagnosis (7–9). Recent studies, most retrospectively and superficial lesions involved, showed that image guided CNB provided sufficient diagnostic reliability to instigate a treatment of lymphoma (10–13). A systemic review revealed that the median rate at which the needle biopsy yielded a subtype specific lymphoma was about 75%, and nearly 25% of CNB of the lymph nodes must be followed by an excisional biopsy to fully classify lymphoma. The cases with inadequate accuracy were due to necrosis, low cellularity, insufficient small sample size, or inappropriate tissue sampling (14). Multi-puncture, Rapid on-site evaluation such as imprint cytology or color Doppler based techniques have been used to guarantee the quality and sufficiency of the sample (15).

Routine B-mode ultrasound was the initial and effective imaging guidance for lymphoma biopsy for decades (16). Color Doppler or power Doppler showed that the angio-architecture of most lymphoma (75%) were highly vascular, potentially guided biopsy procedure and avoided injury of the main vessels neighboring the lesions (17). Contrast enhanced ultrasound (CEUS) is more precise in identifying viable area with vascularity in the lesion, which was confirmed in our previous study which demonstrated CNB of the viable area that verified by CEUS contributed to the increase of histological yield of the anterior mediastinum masses with more cellularity (18). We hypothesized that core needle biopsy of the viable area of the lesion that verified by color Doppler flow imaging (CDFI) and/or CEUS (Viable-targeting US-CNB) would harvest sufficient tissue for the diagnosis of lymphoma. In this retrospective analysis of cohort study, Viable-targeting US-CNB were compared to Routine US-CNB in terms of the yields of clinically actionable diagnosis and complications for the diagnosis of deep-sited dominant lesions suspicious lymphoma, and determinants for the successful US-CNB that established an actionable diagnosis were explored in this study population.

## Patients and Methods

### Ethics Approval, Consent to Participate and Authenticity

This study was a retrospective analysis of a prospective data collection related to a diagnostic test of the approaches of Viable-targeting and Routine US-CNB that established diagnosis for those patients participating eligibility assessment in three lymphoma clinical trials (A2014-052-01, A2016-024-01, and 308-2016-01-01) that registered in the Department of Clinical Research with approval from Institutional Review Board at Sun Yat-sen University Cancer Center (SYUCC). Informed consent was signed before participating the study and undergoing CEUS and US-CNB. The authenticity of this article has been validated by uploading the key raw data onto the Research Data Deposit public platform (www.researchdata.org.cn), with the approval RDD identifier RDDA2019001023.

### Patients’ Identification and Data Collection

Inclusion criteria: All patients were above 18 years old. Clinically suspicious of lymphoma with deep-sited dominant lesion, which was detected by positron emission tomography–computerized tomography (PET-CT) or CT, underwent US-CNB for eligibility criterion of participating clinical trials above. Dominant lesion referred to the lesion or mass with maximum size and/or highest SUV in PET-CT/CT that could be detected by Ultrasound. The axis perpendicular to the probe should be greater than 20 mm.

Exclusion criteria: There was a history of other malignancies. Un-finished pathological diagnosis without required ancillary study followed initial pathological evaluation of the US-CNB sample. Patients lost follow-up.

Patients who signaled willingness to participate the lymphoma clinical trials in the consulting with hematologist and oncologist (ZL, YW, and SW) were assigned to the Viable-targeting group. On the other hand, Patients with reluctance were assigned to the routine US-CNB group that evaluated and guided by gray-scale ultrasonography with CDFI.

All patients underwent Viable-targeting or Routine US-CNB for eligibility criterion of the three lymphoma clinical trials from January 1, 2014 to December 31, 2018. They were identified at the time for eligibility assessment, their related clinical-imaging and pathological data were collected prospectively from the Panoramic Patients Information System from Department of Information at SYSYCC.

### Diagnostic Approaches

Pre-biopsy gray-scale ultrasound, CDFI and CEUS evaluation and US-CNB were performed as described previously (15, 18). CDFI were categorized as not applicable (NA), avascular, minimal, moderate and abundant (19). CEUS was performed with an ultrasonography system (MyLabTwice, Esaote, Genoa, Italy) coupled with a CA541 convex array probe. A 2.4 ml bolus of a US blood pool contrast agent (SonoVue, Bracco, Milan, Italy) was injected into the antecubital vein, followed by a 5-ml saline flush. Next, the deep-sited lesion was scanned continuously for up to 4 min. The dynamic image was recorded on the hard-drive of the ultrasound system. During CEUS, for the deep-sited lesions without background tissue to describe the relative enhancement, it is critical to describe the presence or absence of enhancement and its distribution. And moreover, depiction of enhancement and non-enhancement area are relevant to identify the biopsy target (20).

**FIGURE 1:**
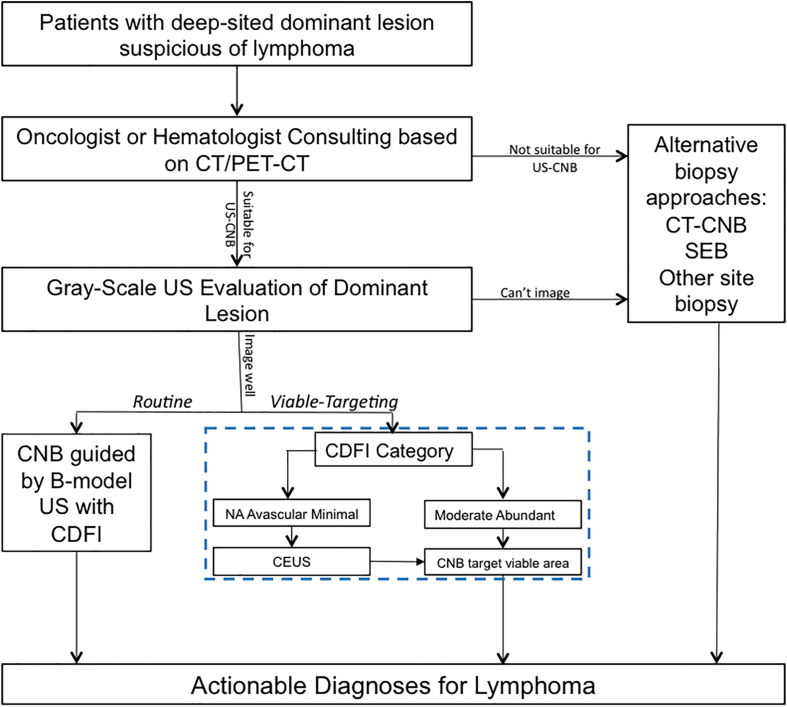
Ultrasound evaluation and guidance of routine or viable-targeting core needle biopsy for suspicious deep-sited lymphoma. US, ultrasound; CDFI, color Doppler flow imaging; CEUS, contrast-enhanced ultrasound; US-CNB, ultrasound guided core needle biopsy; CT-CNB, computerized tomography guided core needle biopsy; SEB, surgical excisional biopsy.

In the approach of Viable-targeting US-CNB, lesions with CDFI category of moderate or abundant were referred to US-CNB directly; Lesions with CDFI categories of NA, avascular, or minimal were referred to CEUS prior to US-CNB. CDFI and CEUS in necessary were employed to evaluate the vascularity of the lesion and to select the targeted area for US-CNB to histologically characterize it in the approach of Viable-targeting US-CNB. Figure 1 shows the ultrasound evaluation and guidance of routine or viable-targeting US-CNB for suspicious lymphoma. All the CNB-related procedures were performed in a free-hand approach by two experienced physicians (JL and JZ, both with over 10 years working experience in ultrasound intervention) with the same ultrasonography system and 18-gauge core biopsy needle (Magnum; Bard, Covington, GA, United States) after routine sterilization and local anesthesia (3–5 ml 1% Lidocaine).

### Reference Standard

The diagnosis of lymphoma was made based on the 2016 WHO Classification of the lymphoid neoplasms. The reference standard was histo-pathological examination with a panel of antibodies including key markers listed in the WHO Classification for lymphoma diagnosis (1). The histological results of core needle biopsy were classified as actionable or non-actionable diagnosis. The actionable diagnosis is referred to a diagnosis (of lymphoma with subtyping) that instigates subsequent therapeutic schedule. Non-actionable diagnosis included unsatisfactory biopsy (necrosis, limited cellularity, sampling error), or a benign disease (fibrous fatty tissue, inflammatory cell infiltration, reactive lymphoid hyperplasia or lymphoproliferative disorder) that was disaccord with the clinical-imaging data, or in terms of suspicious tumor, suspicious lymphoma, undetermined malignancy, or B-NHL without classification for none ancillary study due to insufficient tissue from US-CNB, which all require substantial alternative biopsy approach to establish diagnosis. The establishment of final diagnoses was based on the surgical pathology or response to medical treatment with follow-up at least 6 months (5, 21).

### Sample Size of the Data Collection

The hypothesis that the yield of the actionable diagnosis obtained with Viable-targeting US-CNB resulted in a higher sensitivity than routine US-CNB due to a more viable tissue biopsied. Based on the descriptions of previous studies (14, 18), the estimated sensitivity rate was about 75.0 percent and 90.0 percent at routine US-CNB and Viable-targeting US-CNB, respectively. NCSS Statistical Software (PASS 11.0) was used for computing sample size. To detect more than 15% sensitivity improvement (the superiority test), 194 patients had to be enrolled in this study, with a target alpha of 0.05 and beta of 0.198. Assuming a dropout rate of 10%, the required final sample size was found to be comprised of at least 107 patients in each group.

### Statistics

SPSS software (Version 23.0, IBM, Armonk, NY, United States) was used for statistics. Pearson Chi-square test was used for categorical data; *t*-test was used to compare the quantitative data with normality of distribution that estimated by Kolmogorov-Smirnov test, or Mann-Whitney *U* test was used for the data without normality of distribution. Binary Logistic regression was used to define the key determinants of clinical success of US-CNB for lymphoma diagnosis in this study population. For all tests, *p*-value < 0.05 were considered statistically significant.

## Results

### General

**FIGURE 2:**
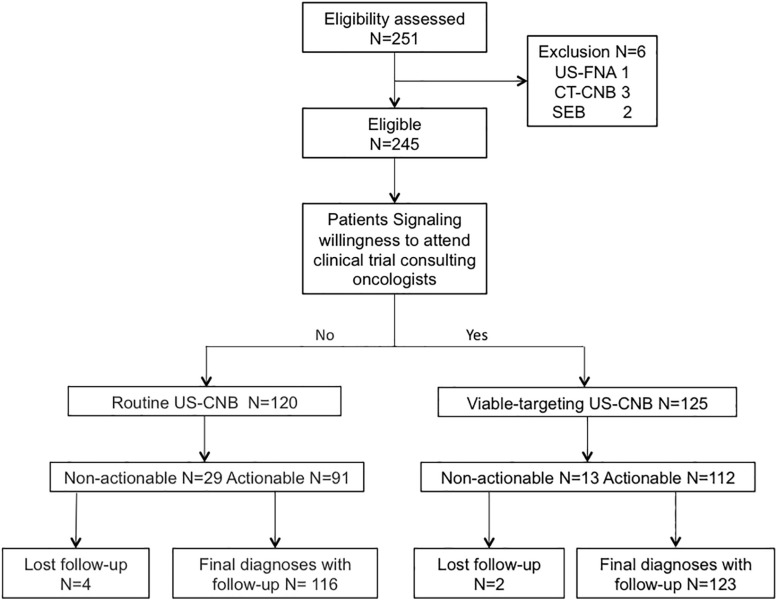
Flowchart of the suspicious lymphoma cases recruited to the viable-targeting and routine ultrasound-guided core needle biopsy groups. US-FNA, ultrasound guided fine needle aspiration; CT-CNB, computed tomography guided core needle biopsy; SEB, surgical excisional biopsy.

A total of 245 patients suspected with lymphoma detecting from CT (*N* = 101) or PET-CT (*N* = 144) were recruited to undergo routine US-CNB (*N* = 120) or Viable-targeting US-CNB (*N* = 125), of which 91 (91/120, 75.8%) and 112 (112/125, 89.6%) showed actionable diagnoses, respectively (*p* = 0.004, OR 0.846, 95% CI: 0.753–0.952). Six patients (6/245, 2.4%) with non-actionable diagnoses were undiagnosed and lost to follow-up. Two hundred and thirty-nine (239/245, 97.6%) patients had final diagnoses with at least 6 months follow-up, The flowchart of the suspicious lymphoma cases recruited to the study groups is illustrated in Figure 2. The patients in both groups were well balanced in regard to the baseline evaluation, which were shown in Table 1.

**TABLE 1:** Patients baseline characteristics of viable-targeting and routine ultrasound guided core needle biopsy groups.

Group	Viable-Targeting CNB *N* = 123	Routine CNB *N* = 116	*p-*value
**Gender**			
Male	72 (58.5%)	70 (61.2%)	0.695
Female	51 (41.5%)	46 (38.8%)	
**Age**, years Median (range)	44.5 (15.0–85.0)	45.3 (18.0–81)	0.653
18–29	31 (25.2%)	23 (19.8%)	0.926
30–59	69 (56.1%)	67 (57.1%)	
≥60	23 (18.7%)	26 (22.4%)	
**Lymphoma History**			
With	33 (26.8%)	32 (27.6%)	0.895
Without	90 (73.2%)	84 (72.4%)	
**Infectious disease**			
No	97 (78.8%)	78 (67.3%)	0.103
HBV	17 (13.8%)	31 (26.8%)	
HCV	2 (1.6%)	0	
EBV	8 (6.3%)	5 (5.2%)	
Syphilis	0	2 (1.7%)	
**Pre-biopsy Imaging**			
CT	54 (43.9%)	43 (37.1%)	0.282
PET-CT	69 (56.1%)	73 (62.9%)	
**Size,** transverse diameter (mm)	52.3 (26.6)	51.3 (30.0)	0.792
longitudinal diameter (mm)	75.5 (32.9)	76.3 (44.5)	0.873
>5 cm of longitudinal diameter	82 (33.3%)	63 (54.3%)	0.051
≤5 cm of longitudinal diameter	41 (66.7%)	53 (45.7%)	
**Diagnosis Phase**			
Initial diagnosis	86 (69.9%)	85 (73.3%)	0.581
Disease progression	17 (13.8%)	11 (9.5%)	
Recurrence	20 (16.3%)	20 (17.2%)	
**Previous biopsy**			
No previous biopsy	58 (47.2%)	61 (52.6)	0.547
Previous CNB of the same lesion	19 (15.4%)	10 (8.6%)	
Previous SEB of the same lesion	1 (0.8%)	2 (0.9%)	
Previous other site biopsy	13 (10.6%)	13 (11.2%)	
Previous lymphoma pathological consulting	32 (26.0%)	30 (25.9%)	
**Repeated procedure**			
No	100 (81.3%)	99 (85.3%)	0.403
Yes	23 (18.7%)	17 (14.7%)	
**Concomitant peripheral biopsy**			
Yes	20 (16.3%)	21 (18.1%)	0.706
No	103 (83.7%)	95 (81.9%)	
**Site of the targeted lesion**			
Mediastinum	31 (25.2%)	27 (23.3%)	0.122
Pleura and lung	6 (4.9%)	3 (2.6%)	
Abdominal cavity*	36 (29.3%)	37 (31.9%)	
Pelvic cavity	18 (14.6%)	7 (6.0%)	
Retro-peritoneum	32 (26.0%)	42 (36.2%)	
**Concurrent Bone marrow biopsy**			
Negative	55 (44.7%)	49 (42.2%)	0.509
Positive	6 (4.9%)	10 (8.6%)	
NA	62 (50.4%)	57 (49.1%)	
**Punctures**			
Range	1–4	1–4	
mean ± SD	2.28 ± 0.504	2.23 ± 0.565	0.455
Total	281	259	
**Pathological Methods**			
H&E staining only	9 (7.3%)	15 (12.9%)	0.211
H&E staining + IHC	61 (49.6%)	61 (52.6%)	
H&E staining + IHC + Molecular	53 (43.1%)	40 (34.5%)	
**MDT**			
Yes	70 (56.9%)	69 (59.5%)	0.561
No	53 (43.1%)	47 (40.5%)	

*Including lesions in liver, spleen; HBV, hepatitis B virus; HCV, hepatitis C virus; EBV, Epstein-Barr virus; CT, computerized tomography; PET-CT, positron emission tomography-computerized tomography; US, ultrasound; CDFI, color Doppler flow imaging; CEUS, contrast enhanced ultrasound; IHC, immuno-histo-chemistry; MDT, multi-disciplinary team; NA, not applicable.

### Comparison of Outcomes Between the Viable-Targeting CNB and Routine CNB Groups

The final diagnoses of lymphoma with subtyping according to the 2016 revision of the World Health Organization classification of lymphoid neoplasms are shown in Table 2. The diagnostic yields of routine US-CNB and viable-targeting US-CNB according to the final diagnosis were 78.4% (91/116) and 91.1% (112/123) (*p* = 0.006, OR 0.554, 95% CI: 0.333–0.920), 14.3% (1/7) and 33.3% (1/3) for benign diseases, 82.6% (90/109) and 92.5% (111/120) for malignancy, 84.0% (84/100) and 93.6% (103/110) for lymphoma, 85.1% (80/94) and 94.2% (98/104) for NHL, 66.7% (4/6) and 83.3% (5/6) for HL, 66.7% (6/9) and 80% (8/10) for other malignancies, respectively.

**TABLE 2:** Final diagnoses with subtypes of the study population from the viable-targeting and routine US-CNB groups.

Diagnosis	Subtyping	Routine group (%)	Viable-targeting group (%)	Total (%)
Non-neoplastic disease	LPD	5 (2.1)	2 (0.8)	7 (2.9)
	TB	2 (0.8)	1 (0.4)	3 (1.3)
Non-Hodgkin’s Lymphoma	BL	5 (2.1)	3 (1.3)	2 (0.8)
	CLL/SLL	0 (0.0)	3 (1.3)	3 (1.3)
	DLBCL NOS	94 (39.7)	46 (19.2)	48 (20.1)
	DLBCL EBV+	1 (0.4)	3 (1.3)	4 (1.7)
	DLBCL-FL	1 (0.4)	5 (2.1)	6 (2.5)
	DLBCL-NMZL	1 (0.4)	0 (0.0)	1 (0.4)
	FL	22 (9.2)	14 (5.9)	36 (15.1)
	GZL	0 (0.0)	2 (0.8)	2 (0.8)
	HGBL	0 (0.0)	3 (1.3)	3 (1.3)
	MALTL	0 (0.0)	3 (1.3)	3 (1.3)
	MCL	0 (0.0)	1 (0.4)	1 (0.4)
	NMZL	3 (1.3)	2 (0.8)	5 (2.1)
	PCM	0 (0.0)	1 (0.4)	1 (0.4)
	PMBL	10 (4.2)	14 (5.9)	24 (10.0)
	AITL	0 (0.0)	1 (0.4)	1 (0.4)
	ALCL ALK+	0 (0.0)	1 (0.4)	1 (0.4)
	ALL	1 (0.4)	0 (0.0)	1 (0.4)
	ENKTL	2 (0.8)	1 (0.4)	3 (1.3)
	PTCL	1 (0.4)	1 (0.4)	2 (0.8)
	Mixed GZL_PTCL	1 (0.4)	0 (0.0)	1 (0.4)
Hodgkin’s Lymphoma	NL	1 (0.4)	0 (0.0)	1 (0.4)
	NS	5 (2.1)	6 (2.5)	11 (4.6)
Mixed	DLBCL_HL-NS	0 (0.0)	1 (0.4)	1 (0.4)
Other Malignancies	BLL	1 (0.4)	0 (0.0)	1 (0.4)
	MM	0 (0.0)	1 (0.4)	1 (0.4)
	TLL	8 (3.3)	8 (3.3)	16 (6.7)
Total	Suspected lymphoma	116 (48.5)	123 (51.5)	239 (100.0)

*US-CNB, ultrasound guided core needle biopsy; TB, tuberculosis; LPD, lymphoproliferative disorder; PCM, plasma cell myeloma; CLL/SLL, Chronic lymphocytic Leukemia/Small Lymphocytic Lymphoma; MALTL, extranodal marginal zone lymphoma of mucosal associated lymphoid tissue; DLBCL EBV+, EBV + DLBCL, NOS; NMZL, nodal marginal zone lymphoma; FL, follicular lymphoma; MCL, mantle cell lymphoma; DLBCL NOS, diffuse large B-cell lymphoma; PMBL, primary mediastinal (thymic) large B-cell lymphoma; BL, Burkitt lymphoma; HGBL, high grade B-cell lymphoma; GZL, gray zone lymphoma: B-cell lymphoma, unclassifiable, with features intermediate between DLBCL and classical Hodgkin lymphoma; T-NHL PLL, T-cell prolymphocytic leukemia; PTCL, peripheral T-cell lymphoma, NOS; AITL, angioimmunoblastic T cell lymphoma; ALCL, anaplastic large-cell lymphoma; ENKTL, extranodal Nk-/T- cell lymphoma, nasal type; HL-NS, nodular sclerosis classical Hodgkin lymphoma; HL-NL, nodular lymphocyte dominant Hodgkin lymphoma; BLL, B-lymphoblastic lymphoma/leukemia; TLL, T-lymphoblastic lymphoma/leukemia; MM, multiple myeloma.*

In Routine US-CNB group, 58 out of 67 (86.6%) lesions categorized with Moderate or Abundant by CDFI were identified as actionable diagnosis; while the rest 49, which were categorized as NA, avascular or minimal, yielded with 34 actionable diagnoses with the percentage of 69.4%.

In Viable-targeting US-CNB Group, 88 lesions that CDFI categorized Moderate or Abundant underwent US-CNB directly, which revealed actionable diagnosis in 85 lesions (96.6%). 35 lesions that CDFI categories of NA, Avascular, or Minimal underwent CEUS evaluation, in which 18 (51.43%) were found overall inhomogeneous enhancement, 16 (45.71%) heterogeneous enhancement with non-perfused regions, and 1 (2.86%) homogeneous enhancement. The subsequent US-CNB revealed 27 (77.14%) actionable diagnoses and 8 (22.86%) non-actionable diagnoses. The CEUS of the lesions with non-actionable diagnoses showed marked (>50% of the size) non-perfused area.

Viable-targeting group found more CDFI categories of moderate or abundant, and had more actionable diagnoses that agreed with the final diagnoses as compared to those of the routine US-CNB group (Table 3).

**TABLE 3:** Comparison of the outcome of viable-targeting core needle biopsy group with routine core needle biopsy group.

Outcome	Viable-Targeting CNB *N* = 123	Routine CNB *N* = 116	*p-*value
**Pre-biopsy US evaluation**			
B mode + CDFI	87 (70.7%)	116 (100.0%)	<0.001
B mode + CDFI + CEUS	36 (29.3%)	0	
**Assigned CNB Diagnoses**			
Non-Actionable	11 (8.9%)	25 (21.6%)	0.006
Actionable	112 (91.1%)	91 (78.4%)	
Benign	1 (0.8%)	1 (0.9%)	
HL	5 (4.1%)	4 (3.4%)	
NHL	98 (79.7%)	80 (69.0%)	
Other Malignancy	8 (6.5%)	6 (5.2%)	
**Final Diagnoses**			
Benign	3 (2.4%)	7 (6.1%)	0.582
HL	6 (4.9%)	6 (5.1%)	
NHL	104 (84.5%)	94 (81.1%)	
Other malignancy	10 (8.1%)	9 (7.8%)	
**Agreement of CNB and Final diagnosis**			
Yes	114 (92.7%)	97 (83.6%)	0.029
No	9 (7.3%)	19 (16.4%)	
**Final Diagnosis Approach**			
Assigned US-CNB	112 (91.1%)	91 (78.4%)	0.009
Repeated US-CNB	3 (2.4%)	10 (8.6%)	
Surgical Excisional Biopsy	3 (2.4%)	7 (6.0%)	
Other Site Biopsy	5 (4.1%)	8 (6.9%)	

*US-CNB, ultrasound guided core needle biopsy; NA, not applicable.*

Minor pain or discomfort was observed in several patients after the procedure of US-CNB and relieved after half an hour observation. No related thoracic or abdominal morbidity such as hemorrhage and pneumothorax were observed during or after the procedure. No seeding of the US-CNB sites were found, excepting one case seeding of trocar wound from the Television video Assisted Surgery observed during the follow-up.

### Clinical and Imaging Characteristics Predict the Yield of US-CNB

Non-actionable diagnoses included pancreatic tissue (*N* = 1), fibrous fatty tissue (*N* = 5), coagulation necrosis (*N* = 3), inflammatory cell infiltration (*N* = 2), reactive lymphoid hyperplasia (*N* = 1), lymphoproliferative disorders (*N* = 2), suspicious tumor, malignancy, or lymphoma (*N* = 10), unclassified hematolymphoid malignancy (*N* = 8), B-NHL without subtyping due to insufficiency of tissue for ancillary study (*N* = 4). Subsequent alterative biopsies of repeated US-CNB (*N* = 13), surgical excisional biopsy (*N* = 10) and other site biopsy (*N* = 13) revealed the actionable diagnoses.

The actionable diagnosis group (*N* = 203) had more blood flow (higher CDFI category) and longer of the transverse-axis of the targeted lesion, increased frequency to the Viable-targeting US-CNB approach and ancillary study, but did not differ in terms of age, gender, disease history, pre-biopsy imaging, disease phase, previous or concurrent superficial biopsy, or anatomical location and the longitudinal axis of the dominant lesion with those of non-actionable group (*N* = 36). In patients with pre-biopsy PET-CT, the actionable diagnosis group demonstrated a higher mean standardized uptake value (SUV) max (Table 4).

**TABLE 4:** Confounding factors related to the actionable diagnoses in ultrasound guided core needle biopsy of deep-sited suspicious lymphoma in the investigated study cohort.

Confounding factors	Actionable diagnoses *N* = 203	Non-Actionable Diagnoses *N* = 36	*p*-value	Odds Ratio	95% C.I.	
					Down	Up
Age (years), mean ± SD	45.7 ± 16.0	40.4 ± 14.9	0.148	1.031	0.989	1.074
Gender, male/female	121/82	21/15	0.387	1.643	0.533	5.063
Cancer History, yes/no	54/149	11/25	0.936	0.918	0.114	7.366
Infectious disease, yes/no	55/148	13/23	0.836	0.871	0.237	3.198
Pre-biopsy imaging, CT/PET-CT	84/119	13/23	0.901	0.922	0.260	3.272
SUV-target, mean ± SD	16.89 ± 8.36	12.88 ± 6.95	0.019	NA	NA	NA
Diagnosis phase, Initial/progress_recurrence	148/55	23/13	0.158	2.615	0.689	9.924
Location, Intra-thoracic/Intra-abdominal	58/145	9/27	0.277	0.784	0.506	1.216
Longitudinal Diameter (mm), mean ± SD	79.4 ± 39.5	56.0 ± 27.7	0.236	0.981	0.951	1.013
Transverse diameter (mm), mean ± SD	54.8 ± 28.6	35.1 ± 18.5	0.034	1.058	1.004	1.115
CDFI category, Avascular, Minimal, NA*/Moderate, Abundant	61/142	23/13	0.042	3.218	0.989	10.464
Repeated procedure, yes/no	27/176	13/23	0.128	0.346	0.088	1.355
Previous biopsy, yes/no	111/92	28/8	0.146	0.694	0.424	1.136
Concurrent Peripheral biopsy, with/without	29/174	12/24	0.586	0.700	0.193	2.533
Concurrent Bone marrow biopsy, Negative/positive/NA	100/11/92	4/5/27	NA	NA	NA	NA
Ancillary studies, with/without*	202/1	13/23	<0.001	7.566	2.966	19.298
MDT, with/without	87/116	12/24	0.527	0.670	0.194	2.315
Groups, Routine*/Viable-targeting	91/112	25/11	0.006	2.603	0.816	8.301

SD, standard deviation; NA, not applicable; CT, computed tomography; PET-CT, positron emission tomography-computed tomography; *The reference for odds ratio of significant confounding factors.

Binary logistic revealed the key determinants for the clinical success of US-CNB to diagnose lymphoma were the Viable-targeting group (*p* = 0.036, OR: 3.560, 95% CI: 1.083–11.702) and ancillary study (*p* < 0.001, OR: 0.007, 95% CI: 0.001–0.036), which relied on the pathologists’ first evaluation of the H&E staining and sufficient amount of the CNB samples. The sensitivity, specificity and accuracy of the constructed model for predicting actionable diagnosis of US-CNB targeting dominant lesion suspicious of being lymphoma were 99.0, 63.9, and 93.7%, respectively.

## Discussion

**FIGURE 3:**
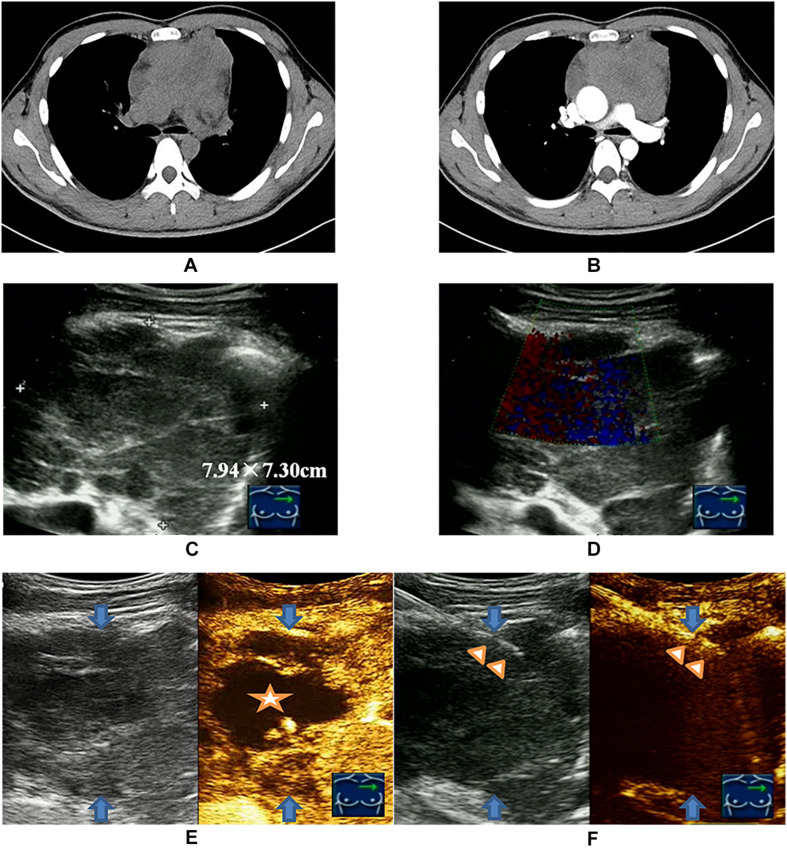
Viable-targeting ultrasound guided core needle biopsy for a 28-year old man with anterior mediastinal mass suspicious of B-NHL with insufficiency of sample for ancillary studies. **(A)** Plain CT scan showed irregular anterior mediastinal mass. **(B)** Contrast enhanced CT showed non-enhanced necrotic area interiorly. **(C)** Ultrasonography showed the anterior mediastinal mass with a size of 7.94 × 7.30 cm by the approach for the parasternal third inter-costal space. **(D)** CDFI was not applicable due to the heartbeat. **(E)** CEUS showed the non-enhanced necrotic area (indicated by the star symbol) and enhanced viable area definitely (peripheral of upper right) of anterior mediastinal mass (blue arrows indicated). **(F)** US-CNB (triangle showed the needle) targeting the enhanced viable area revealed primary thymic large B cell lymphoma of anterior mediastinal mass (blue arrows indicated).

Although the previous studies validated the utility of CNB for diagnosing lymphoma with an accuracy of about 75%, there is still an ongoing debate on the use of core biopsy for the diagnosis of lymphoma, and CNB appeared to be inferior to SEB in providing a definitive diagnosis (14, 22). In the current trend of an increasing use of CNB for lymphoma diagnosis, ultrasound-guided CNB (US-CNB) was regarded as the preferred technique as a front-line diagnostics of lymphoma due to its convenience, mini-invasive approach, real-time application, and cost-effectiveness (10). In the scenarios of imaging detected deep-sited dominant lesion with diffusely enlarged lymph nodes, the presence of peripheral satellite enlarged reactive and/or necrotic lymph nodes may impair the success rate to biopsy these lymph nodes, and core needle biopsy of the deep-sited dominant lesion is necessary (23). The present study developed and implemented an algorithm (blue dotted box of Figure 1) to target the viable portion of the deep-sited lesions for core needle biopsies and demonstrated a diagnostic yield of 91.8% for lymphoma diagnosis, which were superior to that of the Routine US-CNB group (84.0%) and other studies (70–87%) (7, 11). The sensitivity for the actionable diagnoses was significantly higher for viable-targeting group, compared to the routine group, and the sensitivity for the detection of lymphoma with requested ancillary study was significantly better for viable targeting approach based on the precise identifying viable area during US-CNB (Figure 3).

FDG-Avid histology are with great variability in FDG uptake (21). FDG Avidity of the metabolic information obtained from a previous FDG PET/CT scan can have valuable benefits for image guided biopsy (24). A recent study found the mean SUV_max_ of lymph node on PET-CT in confirmatively diagnosed subjects much higher than the deferred counterparts on US-CNB in diagnosing head and neck lymphoma involving cervical nodes (25). Present study selected the deep-site dominant lesion with the maximum SUV for biopsy target, and found that the mean SUV_max_ of the targeted lesion on PET-CT was higher in patients who underwent US-CNB with actionable diagnoses of lymphoma, which imply the lesion with greater SUV should be selected as the target of biopsy. For non- or low-avid disease, further evaluation of the blood supply to the lesion with CDFI and/or CEUS is necessary, as this guides needle placement in the viable portion of the lesion (Supplementary Figure 1).

Some advantages of Viable-targeting US-CNB should be highlighted. First of all, targeting the viable portion of the dominant lesion suspicious of lymphoma verified by CDFI and/or CEUS, based on the recognition that harvesting sufficient viable tissue material for comprehensive immuno-histo-chemical and/or molecular study, was crucial for a definitive lymphoma diagnosis with subtyping. Secondly, large blood vessels detected by CDFI and/or CEUS should not be punctured to avoid complications. Targeting viable area with abundant blood flow signals, and concurrently avoiding injury of the main vessels, were the main reason of low minor complications and no major complications observed in this study. Thirdly, real-time CDFI and/or CEUS should be applied during the procedure of US-CNB to maintain a precise spatial correlation between CNB and CDFI and/or CEUS (Figure 3F). Fourthly, viable-targeting US-CNB has potential benefit of cost-effectiveness from the preliminary analysis (Supplementary Table 1). An emerging effective alternative for mediastinal and abdominal lesions suspicious of lymphoma is endoscopic ultrasound guided biopsy, the diagnostic yield of which can also be improved by the use of CDFI and CEUS (26, 27). But the disadvantages of this technique cannot be ignored. one case of low-grade FL with retro-peritoneum lesion suspicious of residual after four cycles of chemotherapy with the R-CDOP regimen underwent Viable-targeting US-CNB. CEUS revealed a small piece of contrast enhanced portion in the anterior of the lesion, which was targeted by the US-CNB and proved to be the pancreatic tissue by pathologist. This case was a sample error without related abdominal complication. It also implied that, during Viable-targeting US-CNB of greater lesions with surrounding organ compression and movement, especially with pseudo-enlargement after chemotherapy, precisely distinguishing residual disease and normal tissue is necessary for a successful US-CNB.

There were some obvious limitations in this retrospective analysis of the prospective data collection. Firstly, the study population includes patients suspicious of lymphoma examined on CT or PET-CT in a single cancer center, therefore few other benign patients could have been involved. Secondly, although the allocation of Viable-targeting or Routine US-CNB to those patients for eligibility evaluation of the prospective clinical trials were designed in advance, this diagnostic test was not a randomized prospective trial, the pre-biopsy CDFI and/or CEUS were not done randomly but on demand by specialist with signaling willingness from the patients. Thirdly, the overlapping use of CDFI may cause selection bias. Fourthly, the proposed algorithm to target the viable portion of the deep-sited lesions for core needle biopsy is a proposal, and further validation from strictly randomized clinical trial is needed to confirm the findings of this study.

## Conclusion

Although the current guidelines for investigating lymphoma recommend excisional biopsy to obtain ample tissue for architecture assessment and ancillary study to reach an accurate histological classification, the findings of this study showed that viable-targeting US-CNB was superior to Routine US-CNB in term of yield of actionable diagnoses, and could potentially be a regularly performed biopsy approach for the deep-sited suspicious lymphoma lesions.

## Data Availability Statement

The datasets generated for this study are available on request to the corresponding author.

## Ethics Statement

The studies involving human participants were reviewed and approved by the Institutional Review Board at Sun Yat-sen University Cancer Center. The patients/participants provided their written informed consent to participate in this study. Written informed consent was obtained from the individual(s) for the publication of any potentially identifiable images or data included in this article.

## Author Contributions

JL, ZL, and JZ conceived and designed the study. JL, JH, YW, SYW, YM, and JBL prepared and analyzed the data. JX validated the pathological data. JL and JZ drafted and revised the manuscript. YW, JH, SYW, ZL, and JL revised the manuscript. All authors read and approved the final manuscript.

## Conflict of Interest

The authors declare that the research was conducted in the absence of any commercial or financial relationships that could be construed as a potential conflict of interest.

## Supplementary Material

Supplementary Figure 1 A 64-year old woman with systemic lymph nodes enlargement suspicious of lymphoma that previously surgical excisional biopsy of the right inguinal lymph node revealed non-actionable diagnosis of lymphoma with high probability. **(A)** FDG PET/CT revealed systemic lymph node enlargement and the domain lesion with SUV of 8.1 located next to the right external iliac vessels. **(B)** Ultrasonography showed the domain lesion (blue arrow indicated) with size of 24 mm × 37 mm located inferior-medial next to the right external iliac vessel, another neighbor lesion (star symbol showed) with size of 21 mm × 30 mm located upper-medial next to the right external iliac vessel. **(C)** Color Doppler revealed strip-like vessels passed through the both lymph nodes. **(D)** CEUS showed the inferior-medial lesion enhanced with contrast (blue arrow), and no-low enhancement in upper-medial lesion (star symbol showed). **(E)** US-CNB (triangle symbols indicated the needle) targeted the viable inferior-medial lymph node through the non-viable (star symbol) area revealed peripheral T cell lymphoma.
