# Supplementary figures and images for: Core Needle Biopsy Targeting the Viable Area of Deep-Sited Dominant Lesion Verified by Color Doppler and/or Contrast-Enhanced Ultrasound Contribute to the Actionable Diagnosis of the Patients Suspicious of Lymphoma
Source: Front Oncol. 2020 Oct 7;10:500153. doi: 10.3389/fonc.2020.500153 (PMC7577120; doi:10.3389/fonc.2020.500153)

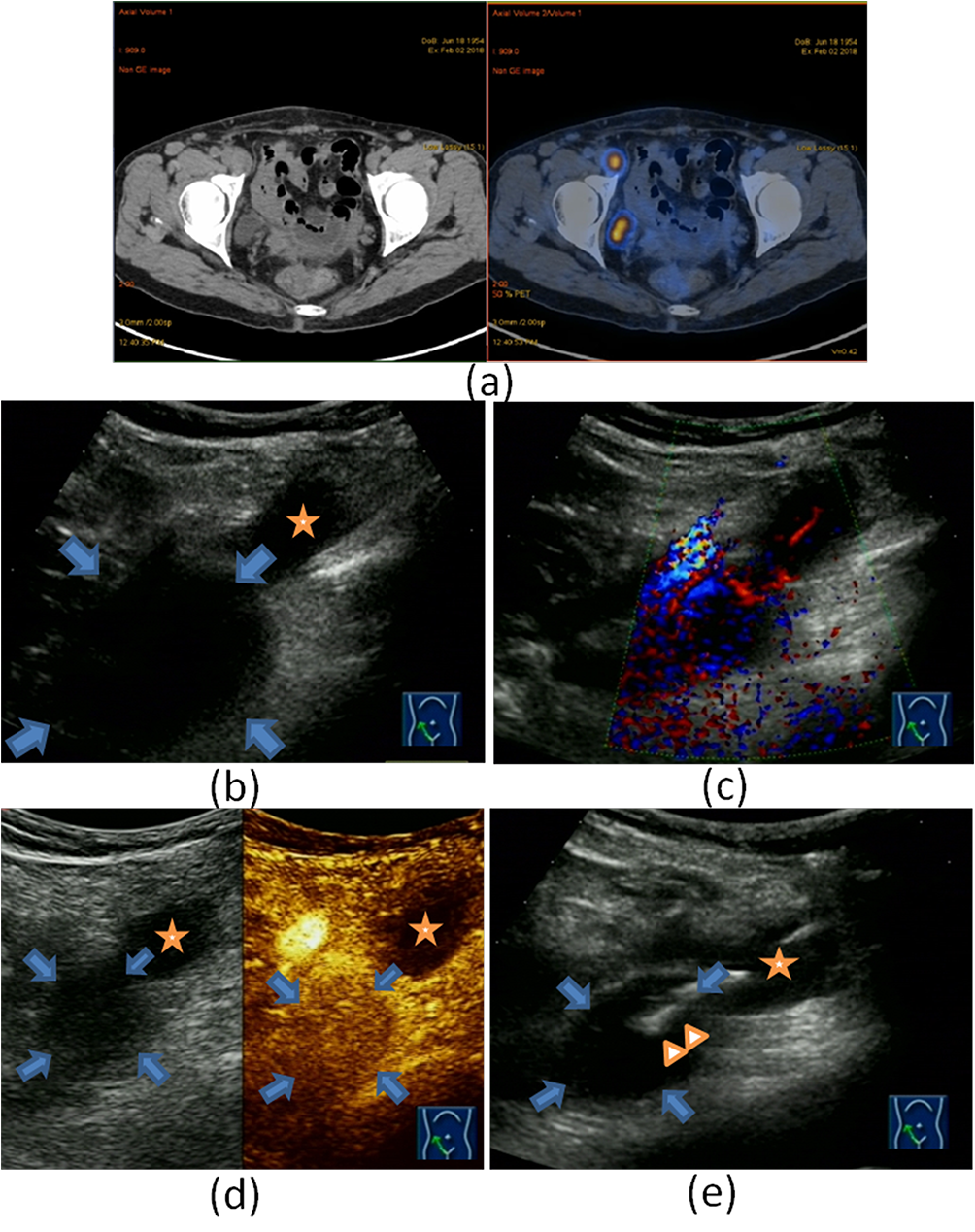

Supplement: Supplementary Figure 1 — A 64-year old woman with systemic lymph nodes enlargement suspicious of lymphoma that previously surgical excisional biopsy of the right inguinal lymph node revealed non-actionable diagnosis of lymphoma with high probability. (A) FDG PET/CT revealed systemic lymph node enlargement and the domain lesion with SUV of 8.1 located next to the right external iliac vessels. (B) Ultrasonography showed the domain lesion (blue arrow indicated) with size of 24 mm × 37 mm located inferior-medial next to the right external iliac vessel, another neighbor lesion (star symbol showed) with size of 21 mm × 30 mm located upper-medial next to the right external iliac vessel. (C) Color Doppler revealed strip-like vessels passed through the both lymph nodes. (D) CEUS showed the inferior-medial lesion enhanced with contrast (blue arrow), and no-low enhancement in upper-medial lesion (star symbol showed). (E) US-CNB (triangle symbols indicated the needle) targeted the viable inferior-medial lymph node through the non-viable (star symbol) area revealed peripheral T cell lymphoma. [file Image_1.TIF]
