# Supplementary material for: Core Needle Biopsy Targeting the Viable Area of Deep-Sited Dominant Lesion Verified by Color Doppler and/or Contrast-Enhanced Ultrasound Contribute to the Actionable Diagnosis of the Patients Suspicious of Lymphoma
Source: Front Oncol. 2020 Oct 7;10:500153. doi: 10.3389/fonc.2020.500153 (PMC7577120; doi:10.3389/fonc.2020.500153)
Supplement: Supplementary file 2 [file Table_1.DOCX]

Supplementary Material

| **Table S1** Comparison of the Cost-effectiveness of Viable-Targeting Core Needle Biopsy Group with Routine Core Needle Biopsy Group | | | |  |
| --- | --- | --- | --- | --- |
| Outcome | Viable-Targeting CNB  N=123 | Routine CNB  N=116 | *p* Value | |
|  |  |  |  |  |
| **Time Between Assigned US-CNB and Actionable Diagnosis**  (days, mean±standard deviation) | 9.14±8.63 | 16.16±31.06 | 0.017 | |
| **Estimated Cost for Actionable Diagnosis**  ($ mean±standard deviation) | 1571.87± 1199.60 | 2318.97± 3562.12 | 0.029 | |
| US-CNB: ultrasound guided core needle biopsy; NA: not applicable | | | |  |

The time (9.14±8.36 days) taken from being assigned US-CNB till the establishment of an actionable diagnosis in Viable-targeting group was lesser than that (16.16±30.06 days) in routine group. Although the cost of a single procedure of routine US-CNB was lower that of Viable-targeting US-CNB due to the addition of CEUS in Viable-targeting approach, the estimated cost per true diagnosis of routine US-CNB ($2318.97±3562.16) was more expensive than that of Viable-targeting US-CNB ($1571.87±1199.00) as more subsequent alterative biopsy approaches were followed in-actionable diagnoses of routine US-CNB. (Table S1)
